# Response to: Comment on “Efficacy of 7-Day and 14-Day Triple Therapy Regimens for the Eradication of* Helicobacter pylori*: A Comparative Study in a Cohort of Romanian Patients”

**DOI:** 10.1155/2016/4760378

**Published:** 2016-08-24

**Authors:** Davide Giuseppe Ribaldone, Giorgio Saracco, Rinaldo Pellicano

**Affiliations:** ^1^General and Specialist Medicine Department, City of the Health and Science of Turin, Bramante Avenue 88, 10126 Turin, Italy; ^2^Oncology Department, University of Turin, Orbassano, 10043 Turin, Italy; ^3^Department of Gastroenterology, Molinette Hospital, University of Turin, 10126 Turin, Italy

In a recent Letter to the Editor [1], Talebi Bezmin Abadi commented on the paper by Arama et al. [2], in which the authors concluded that a 2-week regimen (Proton Pump inhibitor, plus Clarithromycin plus Amoxicillin) is preferable to a 7-day regimen as first-line therapy for* Helicobacter pylori* (*H. pylori*) infection. In his Letter, Talebi Bezmin Abadi concluded that “Certainly, preantibiotic susceptibility tests are inevitable approach in* H. pylori* therapy.” Although we agree with all comments, we found the conclusion inappropriate.

The 4th Maastricht/Florence Consensus Conference recommended abandoning clarithromycin in empirical treatment when the prevalence of resistance is higher than 15–20% [3].

In the paper by Arama et al. [2], the overall eradication rate observed was 70.5%: the number of patients who responded to treatment was significantly greater in the 14-day treatment (84.6%) group compared to patients who received the 7-day treatment (42.3%) (*p* < 0.001).

An acceptable eradication rate is differently defined in literature (>75% [4], ≥ 90–95% [5]) and the clinicians should “use only what works locally” ignoring consensus statements and society guidelines if these are not consistent with local treatment results [6]. Moreover, antibiotic resistance should be considered as a dynamic concept, since its prevalence can change not only among different countries, but also between two different periods in the same area [7].

A culture-based approach, in clinical practice, is often unfeasible and its cost may be hard to afford [8]. Furthermore, the whole process needs a standard of quality (in terms of both materials used for culture and skill of the microbiologist to grow* H. pylori*) that cannot be assured everywhere [7]. Probably, the cost-effectiveness of the culture-based approach may change depending on host-related factors (bleeding, use of certain drugs) and methodology-related factors (number of gastric biopsies, conditions of transport, and laboratory characteristics) [7]. These factors may be favorable in some settings compared to others.

Hence, in the year 2016 a culture-guided treatment must be taken into account only in settings with experience in this issue and after failure of previous regimens.
